# Key Parameters Affecting Kick Start Performance in Competitive Swimming

**DOI:** 10.3390/ijerph182211909

**Published:** 2021-11-12

**Authors:** Ivan Matúš, Pavel Ružbarský, Bibiana Vadašová

**Affiliations:** Faculty of Sports, University of Presov, 080 01 Presov, Slovakia; pavel.ruzbarsky@unipo.sk (P.R.); bibiana.vadasova@unipo.sk (B.V.)

**Keywords:** biomechanics, kinematic analysis, start phase, starting platform

## Abstract

The study aims to determine the contribution of kinematic parameters to time to 5 m without underwater undulating and kicking. Eighteen male competitive swimmers started from three weighted positions and set the kick plate to positions 1–5. We used SwimPro cameras and the Dartfish© software. In the on-block phase, we found significant correlations (*p* < 0.01) between the front ankle angle and block time. The correlations between start phases were statistically significant (*p* < 0.01) between block time and rear ankle angle, respectively, to time to 2 m; rear knee angle and glide time; block time and time to 5 m; time to 2 m and time to 5 m; and flight distance and glide distance. The multiple regression analysis showed that the on-block phase and flight phase parameters, respectively, contributed 64% and 65% to the time to 5 m. The key block phase parameters included block time and rear knee angle. The key flight phase parameters determining time to 5 m included take-off angle and time to 2 m. The key parameters determining the performance to 5 m during the above-water phase include rear knee angle, block time, takeoff angle, and time to 2 m.

## 1. Introduction

The start is of paramount importance in elite competitive sprinting [1], having a significant effect on overall race performance [2,3]. Swimming starts are explosive movements designed to propel athletes through the air as quickly and as far as possible to take advantage of the decreased resistance compared with water [4]. Depending on the event, start times have been shown to consist of between 0.8% and 26.1% of the total race time [5], and the starting performance accounts for 11–12% of the final race time [3].

The swimming start phase of a race is the time from the starting signal to when the center of the swimmer’s head reaches the 15 m mark [4]. The swim start consists of three primary phases that contribute to the total start time [6]. The block phase begins when the swimmer assumes the basic starting position after the “Take your marks” command, followed by the starting signal and the swimmer’s movement on the starting block. This phase ends when the swimmer’s feet leave the block [5]. The flight phase is the time from when the swimmer leaves the block to when the swimmer enters the water. The underwater phase is the time from when the swimmer enters the water to when the swimmer resurfaces to begin free swimming [7]. Swimmers can produce the highest take-off horizontal velocity of approximately 4.48 m/s, which is more than twice the velocity of swimming [8].

Numerous studies have compared various types of swim starts, mainly the track start and grab start, or the track start and kick start [8,9,10,11,12]. With the introduction of the new OSB11 starting platform in 2009, several studies were conducted to determine the effect of using the kick plate on the parameters of start performance [13,14], stance positions in the swim start [15,16], or the key parameters of the swim start [2,17,18,19,20]. However, few studies have dealt with kinematic characteristics of the kick start and times to 5, 10, or 15 m [2,7,15,16,21]. Compared with the grab and track starts, a kick start produces a shorter time to 5 m [19]. Unlike the previous studies, this study addresses the issue of key parameters affecting kick start performance expressed as the time to 5 m in competitive swimming without undulating or kicking underwater motion.

The main purpose of the study was to determine the key parameters affecting kick start performance expressed as the time to 5 m without underwater undulating and kicking. The additional purpose of the study was to identify the contribution of particular start phases to the time to 5 m. These parameters were evaluated using data on the fastest kick starts from OSB12 to the 5 m distance. It was hypothesized that the underwater phase would account for a high degree of variance to kick start performance.

## 2. Materials and Methods

### 2.1. Participants

A total of 18 male competitive swimmers (whose average age, height, and weight were 17.8 ± 1.5 years, 186.2 ± 2.1 cm, and 83 ± 2.5 kg, respectively) participated in this study. The swimmers regularly participated in the Slovak swimming championships, competing in the 50 m freestyle (23.30 ± 0.51 s). Ethical approval for this study was obtained from the Ethics Committee of the University of Presov, Presov, Slovakia (approval No. 1/2021).

When tested, all the swimmers were healthy and did not report any health problems before testing. Each tested person read an information leaflet about the testing and gave his or her written consent.

### 2.2. Test Protocol

The testing session took place in the morning at the Faculty of Sports’ swimming pool facilities at the University of Presov, Presov, Slovakia. Each of the swimmers was informed about the testing conditions. Swimmers first had to determine their preferred starting position on the OSB12 starting block, followed by a warm-up and swimming over the course of 400 m. After the warm-up, 11 waterproof adhesive markers were applied on the swimmers’ bodies [12] in the following locations:

(1) lateral margin of the left transverse tarsal joint,
(2) lateral left and right malleolus,
(3) lateral left and right knee condyles,
(4) left and right greater trochanters,
(5) lateral margins of the left and right scapular spine,
(6) lateral left and right elbow epicondyles,
(7) ulnar styloid processes of the left and right wrist, and
(8) medial side of the 5th metacarpal phalanx joint.

**Figure 1:**
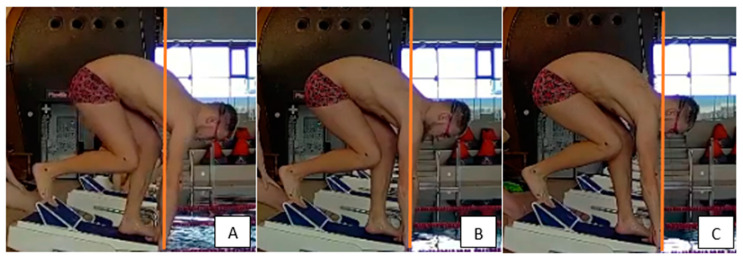
Starting position—block phase: (**A**) front-weighted; (**B**) neutral-weighted; (**C**) rear-weighted.

Then, the swimmers performed three trial kick starts from the OSB12 starting block to become familiar with the three basic starting positions: front-, neutral-, and rear-weighted (Figure 1).

To determine the starting position, we placed a 2 cm-thick bar perpendicular to the starting block’s front edge. The body position in the starting block’s basic position was determined according to the spot marked on the scapular spine as front- (located in front of the bar), neutral- (overlapped with the bar), and rear-weighted (located behind the bar). The swimmers took their marks and responded to a sound signal and an LED light signal at the same time. The swimmers started from starting positions and adjusted the kick plate to positions 1–5. Each swimmer performed three starts from all the three positions (front-, neutral-, and rear-weighted). One kick start trial required 0.7 s. The rest period between starts and changes in the OSB12 kick plate position was 30 s and 2 min, respectively. The rest period after 18 kick start trials was 10 min. The swimmers followed the same order to ensure recovery. Each swimmer performed 45 kick starts over 2 days at the same time of day.

To measure the velocity parameters, a SwimPro camera system was used. The first camera was perpendicular to the starting block at a 0 m distance from the pool’s edge and 1.5 m above the water surface. The second camera was 1.6 m from the pool’s edge and 1.5 m above the water surface. The third camera was 1.6 m from the pool’s edge and 1.7 m below the water surface. The fourth camera was 5 m away from the pool’s edge and 1.7 m below the water surface. To increase the level of lighting, we used halogen and additional LED lights. The camera system was operated at 50 frames per second. We set the shutter speed at 1/1000 s. To evaluate the kinematic parameters for the block, flight, and underwater phases (Table 1), the Dartfish© software (Dartfish ProSuite 4.0, 2005; Fribourg, Switzerland) was used. This software meets the validity and reliability criteria for assessing kinematic parameters using 2D analysis in swimming [22,23]. To determine relationships between kinematic parameters, we used Pearson’s product–moment correlation. Multiple regression analysis was applied to assess the contribution of the kinematic parameters to the 5 m distance. The kinematic parameters were divided into phases—block, flight, and underwater. Regression was calculated for each combination of independent variables using time to 5 m as the dependent variable. The statistical software used was Statistica 12.

## 3. Results

Out of all the weighted starts and kick plate positions, the swimmers produced the shortest times to 5 m when they used the rear-weighted kick start from position 3 on the OSB12 starting block (Table 2).

### 3.1. Relationships between Selected Parameters during Kick Start

#### 3.1.1. Block Phase

The position of the legs on the starting block differed from the basic position on the starting block and the kick plate position. There was a significant relationship (*p* < 0.05) between the front knee angle and the rear knee angle (r = 0.50) and the ankle angle (r = −0.56). There was also a significant relationship between the rear ankle angle and the shoulder position (r = 0.56) as well as block time (r = 0.47). We also found a significant relationship between the hip angle and the shoulder position (r = 0.56). There was a high degree of correlation between the front ankle angle and block time (r = 0.63) (Table 3).

#### 3.1.2. Flight Phase

For the flight phase parameters, there was a single significant correlation (*p* < 0.05) between entry angle and flight distance (r = 0.51) (Table 3).

We also assessed the degree of correlation among the block phase parameters and the flight phase parameters. There was a significant correlation (*p* < 0.05) between the head position at take-off and the front ankle angle (r = 0.48), rear ankle angle (r = 0.50), and shoulder position (r = 0.56). We found a high degree of correlation between (*p* < 0.05) time to 2 m and the rear ankle angle (r = 0.60) as compared with the front ankle angle (*p* < 0.05; r = 0.53). A stronger relationship (*p* < 0.01) was found between the time to 2 m and block time (r = 0.89). There was a significantly negative correlation (*p* < 0.05) between the entry angle and the hip angle (r = −0.49) (Table 3).

#### 3.1.3. Underwater Phase

The correlation analysis of the relationships between the underwater phase parameters did not reveal any significant relationships.

The correlation analysis of the relationships between underwater phase parameters and the basic starting position and movement on the starting block revealed a significant correlation (*p* < 0.05) between the time to 5 m, front ankle angle (r = 0.51), and rear knee angle (r = 0.56). There was a high degree of correlation between the glide time and rear knee angle (r = 0.67) and between the time to 5 m and block time (r = 0.77).

The relationships between the underwater parameters and flight phase parameters were significantly negative between (*p* < 0.05) glide distance and entry angle (r = −0.55). There was a positive correlation between the maximum glide depth and flight time (r = 0.48) and between the time to 5 m and flight time (r = 0.51). Of all the parameters, we found the strongest degree of negative correlation between the glide distance and flight distance (r = −0.99). A strong degree of correlation was found between the time to 2 m and time to 5 m (r = 0.74) (Table 3).

### 3.2. Effect of Start Phases on the Time to 5 m

We applied multiple regression analysis to determine saturation of the dependent variable (time to 5 m) and the independent variables in particular phases of the start.

#### 3.2.1. Block Phase

The initial phase of start forms the basis of a swim start, affecting all subsequent start phases. As the multiple regression analysis shows for the time to 5 m and block parameters, the block time is the most relevant (b* = 0.74) in the regression model, followed by the rear knee angle (b* = 0.65). These values were also statistically significant. According to the model, there was a 64% variability in the time to 5 m. The time to 5 m extended if the swimmer produced a longer block time and a greater rear knee angle (Table 4).

#### 3.2.2. Flight Phase

The flight phase follows the take-off from the starting block. We used the multiple regression analysis to determine the relationship between the time to 5 m and the flight phase parameters. The analysis (Table 5) shows that the time to 2 m (b* = 0.69) is the most relevant in this model, followed by the take-off angle (b* = 0.42), which was also statistically significant. According to the model, there was a 65% variability in the time to 5 m. We found that the time to 5 m extended if the swimmer produced a longer time to 2 m and a greater take-off angle (Table 5).

#### 3.2.3. Underwater Phase

Using the final model of the multiple regression analysis, the relationship between the time to 5 m and underwater phase parameters was determined. None of the variables were statistically significant. The model showed a low percentage of variability in the time to 5 m (Table 6).

## 4. Discussion

In this study, the key parameters underlying the swim start performance in our competitive swimmers were identified. Because the 5 m distance was studied, the study dealt with the efficiency of take-off from the OSB12 starting block to 5 m without kicking or undulating or the first swimming movements. The swimmers completed 45 kick starts, i.e., three starts from each kick plate position, changing the shoulder position in the basic starting position (front-, neutral-, and rear-weighted). The results showed that the swimmers produced shorter times to 5 m when they used the rear-weighted kick start from position 3 on the OSB12 starting block. This study dealt with all the phases of the swim start, although some studies [5,25] have highlighted the importance of the underwater phase. The analysis of the block phase of the start showed that the front ankle angle, rear knee angle, and block time were significantly correlated with the time to 5 m. The highest degree of correlation was found between block time (*p* < 0.01) and the time to 5 m. Other authors, who also used correlation analysis, reported similar findings [9,17,26,27,28,29,30], reporting high correlations with the times to 5, 10, and 15 m. There was a specific type of correlation between block time and leg position, which affects the movement on the OSB12 starting block. The strongest relationship was found for the front ankle angle. Slawson et al. [31] found significant positive correlations between the peak force values and the rear knee angle produced in both horizontal and vertical directions (r = 0.701 and 0.688).

The athletes performed better starts when they adopted a high front knee angle of 135–14° and rear knee angle of 75–85° at set-up. In this study, when the shortest times to 5 m were produced, the front and rear knee angles were 133° and 80°, respectively. The relationships between the distance to 5 m and the parameters of the basic starting position and movement on the starting block showed that this phase accounted for a 64% variability in the time to 5 m. The key parameters include the block time and rear knee angle. Because all other phases of the start depend on this phase, the phases should be studied in more detail. A shorter reaction time results in a shorter time to 15 m [32]. Some studies show that there must be a balance between block time and horizontal velocity [24]. In a study by Matúš et al. [33], swimmers with the shortest block times did not produce the shortest times to 5 m. Swimmers produced the shortest block times when using the front-weighted kick start. However, the shortest times to 5 m were found for the rear-weighted kick start. When the shortest times were recorded, the kick plate was set to position 3 [34]. Shorter block times may be caused by improved anticipation of the start signal and increased strength and take-off performance from the staring block [32]. For instance, Vilas-Boas et al. [35] found that track start produced higher impulses on the starting block, which led to higher horizontal velocities at both take-off and water entry. Consequently, shorter reaction times may negatively affect these values. Research studies by Matúš et al. [33,34] aimed at the grab, track, or kick start or their modifications showed that the swim start during which swimmers produced shorter reaction times did not result in shorter times to 5, 7.5, and 10 m, respectively. In general, we conclude that production of shorter reaction times without losing horizontal velocity may be achieved by using a new OSB starting block. When used, the rear foot is placed upon the kick plate, eliminating the loss of velocity in the horizontal direction [8]. The abovementioned facts show that swimmers should focus on producing the shortest reaction times possible without losing horizontal take-off velocity.

Resistance is lower during the flight phase than after water entry [24,26]. Therefore, swimmers should optimize this phase. The analysis of the flight phase parameters showed a statistically significant correlation between the time to 5 m and flight distance and between the time to 5 m and the time to 2 m. The correlation between the time to 5 m and the time to 2 m was higher. Some studies have pointed to the relationships between the flight distance, take-off angle (r = −0.59 [36]; r = 0.88 [37]), and reaction time (r = 0.36, [13,38]). There was a statistically significant correlation between the angle at take-off and the angle at hand entry (r = 0.57, [22]). In this study, of the flight phase parameters, statistically significant relationships (*p* < 0.05) were found only between the take-off angle and flight time. Additionally, we found significant relationships between flight phase parameters and block parameters. The highest number of correlations was found between the head position at take-off from the starting block and legs, shoulder position, time to 2 m, and ankle angles. The highest degree of correlation (*p* < 0.01) was found between the reaction time and the time to 2 m. When determining the time to 5 m and flight phase parameters, this phase is explained by the 65% variability of the time to 5 m. The key parameters in particular include the time to 2 m and the take-off angle.

Results of numerous studies have confirmed that the underwater phase is an important phase that determines the start performance [2,24,39,40] because advantages gained above the water surface (e.g., higher horizontal take-off velocity) are transferred to the underwater phase. Our analysis of the underwater phase parameters in this study showed that there were no significant correlations between glide time, glide distance, maximum depth, and time to 5 m. However, significant correlations between the first two phases and the underwater phase have been shown [24]. In this study, we found a correlation between the underwater phase and the first phase of the start. There was a significant correlation (*p* < 0.01) between the glide time and the rear knee angle. Entry angle, flight distance, and glide distance significantly correlated with the flight phase parameters. A higher degree of correlation (*p* < 0.01) was found between flight distance and glide distance. The maximum depth was −0.90 m, which corresponds with the recommendations by Tor et al. [2] who dealt with the optimization of underwater trajectories. According to their recommendations, swimmers should achieve a maximum depth of approximately −0.92 m to minimize the velocity lost during the underwater phase. This variable significantly correlated (*p* < 0.01) with flight time. During the underwater phase, the time to 10 m, the time underwater in descent, and the time underwater in ascent have been shown to account for 96% of the variance in start time [41]. In this study, the determination of the time to 5 m by the underwater parameters (glide time, glide distance, and maximum depth) explained the low percentage of variability. We assume that the low percentage may have been caused by the resulting distance. For instance, the final race time was highly determined by the underwater phase parameters for the 15 m distance [2]. The time length of the first phases of the start is very short compared with the underwater phase to 15 m. Therefore, it is logical that there is a high degree of determination by the underwater phase parameters [2]. Upon watching the recorded videos, we found that the swimmers achieved the maximum depth at the 5 m distance during descent or at the end of descent. According to the proposed recommendations [7,42], swimmers should stop gliding after 5.5–6.5 m. Therefore, in this study, gliding without kicking or undulatory movement was assessed. Of note, the underwater phase and final performance may affect the timing of the first kick, hydrodynamics, and underwater kicking ability [2,43].

## 5. Conclusions

The key parameters of the kick start that contribute to the start performance to the 5 m distance were identified. Of all the weighted starts and kick plate positions, our swimmers produced the shortest times to 5 m when they used the rear-weighted kick start from position 3 on the OSB12 starting block. In the block phase, there was a significant relationship between the front and rear leg parameters, and the front leg had a more significant effect on the block time. In the flight phase, there was a single significant correlation between entry angle and flight distance. There was a significant correlation between front leg and rear leg parameters and the time to 2 m. The highest degree of correlation was found between the rear ankle angle, the block time, and the time to 2 m. In the underwater phase, there was a significant correlation between the rear knee angle and glide time and between the block time and the time to 5 m. The relationships between the underwater phase parameters and the flight phase parameters showed a high degree of correlation between flight distance and glide time and between the time to 2 m and the time to 5 m. According to our multiple regression analysis model, the block phase contributed 64% to the time to 5 m. The key block phase parameters that determined the time to 5 m included the block time and the rear knee angle. Flight phase parameters contributed 65% to the time to 5 m. The key flight phase parameters that determined the time to 5 m included the take-off angle and the time to 2 m. The underwater phase contributed to a small extent to the time to 5 m. The key parameters determining the performance to 5 m during the above-water phase included the rear knee angle, block time, take-off angle, and the time to 2 m.

## Figures and Tables

**Table 1:** Description of the kick start parameters.

*Block Phase*			*Definition*	*Authors*
Front knee angle	FKA	(°)	Hip/ankle at the set positon	[11,13]
Front ankle angle	FAA	(°)	Knee/ankle/finger toe at the set position	[11,13]
Rear knee angle	RKA	(°)	Hip/ankle at the set positon	[11,13]
Rear ankle angle	RAA	(°)	Knee/ankle/finger toe at the set position	[11,13]
Hip angle	HA	(°)	Ankle/hip/shoulder	[22]
Shoulder position	SP	(°)	Shoulders in front of/above/behind hands	[16]
Block time	BT	(s)	Starting signal—feet separation from the platform	[11,13,14,24]
*Flight phase*				
Take-off angle	TA	(°)	Ankle/hip/horizontal	[22]
Take-off head position	HP	(m)	Water surface/head	-
Time to 2 m	T2	(s)	Starting signal/head cross the 2 m	-
Entry angle	EA	(°)	Horizontal/fingertips/hip joint	[15]
Flight time	FT	(s)	Take-off/hand entry	[11,13,14,24]
Flight distance	FD	(m)	Take-off/hands touch the water	[11,13,14,24]
*Underwater phase*				
Glide time	GT	(s)	Hand entry/head cross the 5 m	
Glide distance	GD	(m)	Hands touch the water/head cross the 5 m	
Maximal depth	MaxH	(m)	Head reaches the maximum depth	[2]
Time to 5 m	T5	(s)	Starting signal—head cross the 5 m	

Note: °—degree; m—meter; s—second.

**Table 2:** Kinematic parameters for the rear-weighted kick start from position 3 on the OSB12 starting block—the shortest time to 5 m.

	*FKA*	*FAA*	*RKA*	*RAA*	*HA*	*SP*	*BT*	*TA*	*HP*	*T2*	*EA*	*FT*	*FD*	*GT*	*GD*	*MaxH*	*T5*
	(°)	(°)	(°)	(°)	(°)	(°)	(s)	(°)	(m)	(s)	(°)	(s)	(m)	(s)	(m)	(m)	(s)
*M*	133.2	128.4	79.5	96.6	44.7	5.7	0.79	40.6	1.3	1.05	37.5	0.35	2.73	0.55	2.27	−0.90	1.70
SE	1.4	0.9	1.1	1.7	1.0	0.5	0.06	1.5	0.0	0.05	0.9	0.03	0.11	0.04	0.11	0.02	0.08

Note: FKA—front knee angle; FAA—front ankle angle; RKA—rear knee angle; RAA—rear ankle angle; HA—hip angle; SP—shoulder position; BT—block time; TA—take-off angle; HP—take-off head position; T2—time to 2 m; EA—entry angle; FT—flight time; FD—flight distance; GT—glide time; GD—glide distance; MaxH—maximal depth; T5—time to 5 m; °—degree; m—meter; s—second.

**Table 3:** Correlations between swim start parameters.

		Block Phase							Flight Phase						Underwater Phase			
		FKA	FAA	RKA	RAA	HA	SP	BT	TA	HP	T2	EA	FT	FD	GT	GD	MaxH	T5
*Block phase*	FKA	1.00																
	FAA	0.18	1.00															
	RKA	0.50 *	0.43	1.00														
	RAA	−0.56 *	0.15	0.01	1.00													
	HA	−0.27	0.03	0.35	0.42	1.00												
	SP	−0.28	0.11	0.22	0.56 *	0.56 *	1.00											
	BT	−0.04	0.63 **	0.29	0.47 *	0.29	0.24	1.00										
*Flight phase*	TA	0.31	0.05	0.23	0.25	0.23	0.34	0.22	1.00									
	HP	0.10	0.48 *	0.50 *	0.41	0.30	0.56 *	0.24	0.36	1.00								
	T2	−0.24	0.53 *	0.31	0.60 **	0.36	0.33	0.89 **	0.04	0.36	1.00							
	EA	0.18	0.16	−0.08	−0.28	−0.49 *	−0.06	−0.31	−0.32	0.00	−0.43	1.00						
	FT	−0.23	0.17	−0.05	0.26	−0.14	0.05	0.25	0.21	0.26	0.38	−0.20	1.00					
	FD	0.21	0.19	−0.05	−0.28	−0.33	−0.23	−0.16	0.03	0.08	−0.21	0.51 *	0.38	1.00				
*Underwater phase*	GT	0.35	−0.04	0.67 **	−0.05	0.15	0.12	−0.11	0.22	0.15	−0.12	−0.09	−0.16	−0.41	1.00			
	GD	−0.21	−0.22	0.03	0.29	0.39	0.26	0.15	0.00	−0.10	0.21	−0.55 *	−0.41	−0.99 **	0.36	1.00		
	MaxH	−0.31	−0.04	−0.18	0.19	0.07	0.17	−0.15	−0.01	0.30	−0.02	−0.09	0.48 *	−0.10	0.05	0.06	1.00	
	T5	0.06	0.51 *	0.56 *	0.43	0.24	0.26	0.77 **	0.37	0.36	0.74 **	−0.36	0.51 *	−0.18	0.41	0.14	0.12	1.00

Note: *—significant at *p* < 0.05; **—significant at *p* < 0.01; FKA—front knee angle; FAA—front ankle angle; RKA—rear knee angle; RAA—rear ankle angle; HA—hip angle; SP—shoulder position; BT—block time; TA—take-off angle; HP—take-off head position; T2—time to 2 m; EA—entry angle; FT—flight time; FD—flight distance; GT—glide time; GD—glide distance; MaxH—maximal depth; T5—time to 5 m.

**Table 4:** Regression analysis for the block phase.

Multiple R = 0.89, R Squared = 0.79, Adjusted R Squared = 0.64, F (7.10) = 5.37, *p* < 0.00, Standard Error = 0.05						
*n* = 18	b*	SE b*	b	SE b	t(10)	*p*-Value
Intercept			1.82	3.17	0.57	0.58
FKA	−0.21	0.24	−0.01	0.01	−0.87	0.41
FAA	−0.21	0.21	−0.02	0.02	−0.99	0.35
RKA	0.65	0.24	0.05	0.02	2.76	0.00
RAA	0.10	0.24	0.00	0.01	0.40	0.70
HA	−0.31	0.21	−0.02	0.02	−1.45	0.18
SP	0.02	0.20	0.00	0.03	0.12	0.91
BT	0.74	0.23	1.02	0.31	3.30	0.00

Note: FKA—front knee angle; FAA—front ankle angle; RKA—rear knee angle; RAA—rear ankle angle; HA—hip angle; SP—shoulder position; BT—block time; b*—standardized regression coefficient; b—non-standardized regression coefficient.

**Table 5:** Regression analysis for the flight phase.

Multiple R = 0.88, R Squared = 0.78, Adjusted R Squared = 0.65, F (6.11) = 6.34, *p* < 0.00, Standard Error = 0.05						
*n* = 18	b*	SE b*	b	SE b	t(11)	*p*-Value
Intercept			−0.18	1.10	−0.17	0.87
TA	0.42	0.18	0.02	0.01	2.39	0.00
HP	−0.11	0.18	−0.58	0.91	−0.64	0.54
T2	0.69	0.19	1.03	0.29	3.58	0.00
EA	0.35	0.22	0.03	0.02	1.56	0.15
FT	0.40	0.20	0.95	0.47	2.03	0.07
FD	−0.37	0.21	−0.27	0.16	−1.75	0.11

Note: TA—take-off angle; HP—take-off head position; T2—time to 2 m; EA—entry angle; FT—flight time; FD—flight distance; b*—standardized regression coefficient; b—non-standardized regression coefficient.

**Table 6:** Regression analysis for the underwater phase.

Multiple R = 0.42, R Squared = 0.18, Adjusted R Squared = 0.002, F (3.14) = 1.01, *p* < 0.416, Standard Error = 0.08						
*n* = 18	b*	SE b*	b	SE b	t(14)	*p*-Value
Intercept			1.8	1.06	1.70	0.11
EA	0.41	0.26	0.73	0.47	1.57	0.14
FT	−0.01	0.26	−0.09	0.19	−0.04	0.97
FD	0.10	0.24	0.45	1.06	0.43	0.68

Note: EA—entry angle; FT—flight time; FD—flight distance; b*—standardized regression coefficient; b—non-standardized regression coefficient.

## Data Availability

The datasets generated and analyzed for this study can be requested by correspondence authors.
